# Ligation of Intersphincteric Fistula Tract Is Suitable for Recurrent Anal Fistulas from Follow-Up of 16 Months

**DOI:** 10.1155/2017/3152424

**Published:** 2017-02-08

**Authors:** Yansong Xu, Weizhong Tang

**Affiliations:** ^1^Emergency Department, The First Affiliated Hospital of Guangxi Medical University, Nanning 530021, China; ^2^Colorectal and Anal Department, The First Affiliated Hospital of Guangxi Medical University, Nanning 530021, China

## Abstract

Since 2007, ligation of the intersphincteric fistula tract (LIFT) for the management of anal fistula was all introduced with initial success and excitement. It remains controversial which surgical procedure is suitable for transsphincteric fistula, especially to complex anal fistula. This retrospective study was designed to evaluate the results in patients with recurrent anal fistula by LIFT. A retrospective study of 55 complex fistula patients who underwent LIFT procedure in a single medical center was analyzed. Patients and fistula characteristics, complications, and recurrences were reviewed. All 55 patients underwent the procedure with a median follow-up of 16 months. Median operative time was 44 (range 23–88) minutes. Of the 55 patients, 33 (60%) healed completely and did not require any further surgical treatment at end of follow-up. Twenty-two (40%) recurrences and six complications were observed. Compared with patients who had undergone more than two surgical procedures, LIFT was more suitable for patients who had undergone one to two surgical procedures, and significant difference was observed in number of operations before LIFT (*p* = 0.002). Clinicians can consider the use of LIFT for the treatment of recurrent anal fistulas. A larger number of patients and prospective study are needed to be performed.

## 1. Introduction

Complex anal fistula has been a hot topic in clinic. Many surgical techniques have been described for the treatment of such anal fistula, including the use of seton, fibrin glue, collagen plugs, rectal advancement flaps, fistulotomy with sphincter repair, and rerouting the fistula tract [1]. However, the results have been variable, and no one procedure is superior to the others absolutely. It is worth our concern that the goal of any treatment procedure is to obliterate the tract and to have low recurrence rates while maintaining full continence. A fistulotomy lays open the fistulous tract, thus leaving smaller unepithelized wounds, which hastens the wound healing. Until now, fistulotomy is still the most widely used. But, the high cure rate is limited by the fact that laying open a transsphincteric fistula tract and cutting both the internal and the external sphincter has the risk of fecal incontinence ranging up to 40% [2]. In 2007, Rojanasakul et al. [3] described a new therapeutic option for cases like these, with very promising initial results. Since then, LIFT has been used as a sphincter-sparing technique to repair anal fistulas, because of early satisfactory results. Literature reports showed the rate of primary healing in patients ranging from 40% to 95% [4, 5]. The aim of this study is to present a retrospective study in LIFT procedure in treatment of recurrent anal fistulas.

## 2. Materials and Methods

A retrospective analysis was performed of consecutive patients who underwent the LIFT procedure at First Affiliated Hospital of Guangxi Medical University. Cryptoglandular anal fistulas arise from an inflammation of the proctodeal glands, which in humans are only rudimentary and are situated in the intersphincteric space. Patients who were diagnosed with recurrent anal fistulas after other procedures were collected. Recurrent anal fistulas included those who had undergone at least 1 operation before LIFT. We diagnosed anal fistula through the ultrasound examination. One female patient was misdiagnosed as perianal abscess and underwent incision and drainage, but 6 months after operation, there was a rectovaginal fistula. According to Continence Grading Scale, we classified the preoperative anal incontinence. Seven patients underwent incontinence before LIFT (Table 1). Patients with Crohn's disease and tuberculosis were diagnosed by endoscopy, pathology, and imaging, and all patients did not receive medication before operation. LIFT was performed by a single surgeon.

### 2.1. Surgical Procedure and Follow-Up

All patients had a full bowel preparation with oral lavage solution before operation. All patients did not receive preoperative antibiotics. The patients were placed in the prone jackknife position with the buttocks taped widely apart. Epidural anesthesia or subarachnoid anesthesia was used based on patient and anesthesiologist preference. The details of the LIFT procedure have been described in a previous study from Rojanasakul et al. [3]. And the basic steps are as follows. (1) A probe is maneuvered from the external opening to internal through the fistula tract. The skin over the intersphincteric groove is marked, with the probe in place. (2) Using blunt dissection in the intersphincteric plane, the internal and external sphincter muscles were separated to expose the fistula tract. (3) Care is taken not to divide any sphincter muscle. Once the tract is dissected free, it is encircled and the probe can be removed. (4) Next, the fistula tract is divided and ligated. The incision was closed with absorbable sutures after the wound was irrigated. The external opening was left open to drain. All patients received antibiotics after operation. Broad-spectrum II antibiotics (Cefathiamidine) and antianaerobic were used for 2 days after surgery. All the patients routinely used Potassium Permanganate and benzalkonium chloramine to clean perianal wounds.

The patients were followed up and monitored for complications, recurrence in the clinic. Recurrence was defined as a nonhealing wound or reappearance of an external opening with persistent discharge or reappearance of a fistula after the initial wound had healed. Follow-up appointments were scheduled at 2 weeks after surgery unless it was required sooner per patient symptoms. Clinical healing was defined as the absence of fistula drainage with no evidence of residual fistula tract, with closure of the internal opening on anoscopy, closure of the external opening and intersphincteric groove wound on examination, and no evidence of abscess formation at any time during follow-up.

### 2.2. Statistical Analysis

Statistical analysis was performed using SPSS 16.0. *T* tests were used to compare continuous variables, and Fisher exact tests were used for comparison of proportions. All *p* values were two-sided and considered statistically significant where *p* ≤ 0.05 (GraphPad Software, Inc., La Jolla, CA).

## 3. Results

In this retrospective study from 2011 to 2014, 55 patients with recurrent anal fistulas who had undergone at least 1 operation were selected. Of 55 patients, 35 were male and 20 were female. The age ranged from 17 to 62 years, with a mean of 46 years. According to classification based on the types of anal fistula, the proportion of transsphincteric anal fistula was significantly higher than other types (Table 2). Preoperative and postoperative incontinence scores were assessed using the Cleveland Clinic Florida Fecal Incontinence score (CCF-FI) [6]. Thirty-six patients in perianal pain, ten in perianal infection, fifteen in itch, five in gas incontinence, and two in fecal incontinence. Comorbidities were presented in 32.7% (18/55) of patients and included hypertension, diabetes mellitus, Crohn's disease, and tuberculosis (Table 2). The median operating time was 44 (23–88) minutes. There were no intraoperative complications. Six postoperative complications were observed. One patient with rectovaginal fistula developed an anal fissure. Two patients with horseshoe fistula presented with persistent anal pain. One patient developed fecal incontinence (≤1/month) and the other presented gas incontinence (≥1/week). One patient with perianal infection was cured. The postoperative hospitalization time ranged from 1 to 4 days, with a mean of 2 days (Table 3).

Outpatient follow-up was taken, with a mean follow-up of 16 months. Of the 55 patients in the study, 33 patients (60%) healed successfully after their LIFT procedure and 22 relapsed (40%). Of the 22 recurrences, 15 patients received more than two operations before LIFT. Patients who have underwent less than two previous operations (78% healing rate) had more higher healing rate than those who had underwent 2 or more operations before undergoing LIFT (36% healing rate) at end of follow-up (median, 16 months). Compared with patients who had undergone more than two surgical procedures, LIFT was more suitable for patients who had undergone one to two surgical procedures, and significant difference was observed in number of operations before LIFT (*p* = 0.002, Table 4).

## 4. Discussion

First paper concerning the LIFT technique was described in 2007 by Rojanasakul and his colleagues and reported impressive healing rate (over 94%) with no complications for transsphincteric fistulas. Since then, LIFT was familiar to clinicians; long-term success rates from studies with a follow-up period report healing rates of 40–95% for LIFT (Table 5). Of the 55 LIFT procedures performed in our series, the main finding of this study is overall healing of 60% at a median follow-up of nearly 16 months. The strength of this study is that it represents the first description of the single-institution experience with this technique and is the largest published recurrent fistula of patients who have underwent the LIFT procedure. This makes the case for LIFT as an excellent choice for recurrent anal fistulas in future. The average operative time ranged from 10 minutes to 35 minutes in other reports [4, 7, 8]. The present research collected recurrent anal fistulas. With the increase of the number of operations, the difficulty of LIFT increased, and the time would be extended.

Multifactors affected the healing rate of anal fistula, including possible complexity of the original fistula, manipulation of operative bed, comorbidities, the surgeon's proficiency with the procedure, previous operations, and other unidentified factors. Abcarian et al. [9] reported that the patients with one previous surgery had a healing rate of 75%, and the patients with two or more previous surgeries had a success rate of 65% at end of follow-up (median, 9 months). Our result is similar to the finding of Abcarian et al. and shows that healing rates are better for patients who have underwent less than two previous operations (60% healing rate) versus those who had underwent 2 or more operations before undergoing LIFT (36% healing rate) at end of follow-up (median, 16 months). Murugesan et al. [10] have provided a narrative synthesis of the findings from the 22 studies; no incontinence or change in continence were reported in 18/21 studies analyzed. In this retrospective study, two incontinences were observed, including gas and fecal incontinence. We also found that, although the majority of LIFT recurrence presented early, some occurred beyond 6 months and as late as 12 months after the initial procedure. Hence, the time of follow-up cannot be ignored. Currently published median follow-up ranges from 5 to 9 months, but several authors have found that late recurrences can occur 7 to 8 months after surgery procedure [11–13]. Thus, the short-term observation may be an overestimate of the success rate. Even if the external and internal orifice is healed and there is no gas or liquid present in the track on ultrasound, it is still possible for incomplete closure presumably with a risk for recurrence. The reasons for the high success rate of the present study may be related to short-term follow-up. Follow-up extended to two or more years should clarify this point [8]. Extended follow-up is needed to better understand the long-term outcome of LIFT. According to other literatures and this study, we suggest extended follow-up of at least 12 months after initial surgery to be certain that complete healing has occurred.

Although LIFT has obvious advantages in treatment of complex anal fistula surgery, surgical injury is difficult to avoid, especially for multiple postsurgical patients. Several reasons can explain complications. Firstly, although we have a wealth of clinical experience, the postoperative care cannot be ignored; we believe that postoperative complications associated with care. Secondly, we only used antibiotics for two days, and appropriate extension of antibiotic use time might reduce postoperative complications. Thirdly, there was no professional medical staff to guide patients to wash the wound after discharge. Of the 22 recurrences, there were 3 patients with diabetes mellitus, 3 patients with IBD including 2 with Crohn's disease and 1 with tuberculosis, and other cases. These risk factors could potentially be optimized by asking patients to treat diabetes mellitus and IBD before undergoing surgery. Technical factors may be a factor, in order to reduce a possible bias for treatment failures, and LIFT procedure was performed by one skilled surgeon in this study. Hence, this factor may increase healing rates in either LIFT procedure or other procedures.

Limitations to this study included its nonrandomized, small samples, short follow-up, and nonprospective design. Follow-up proved challenging, particularly in patients who healed because they tend to miss appointments when feeling well. The small sample size did not allow for multivariate analysis. The cohort was somewhat heterogeneous because there were patients with long-standing fistulas as well as previous procedures.

Our study is a retrospective single-institution study which lacked adequate power to determine differences in patient preoperative variables such as previous operative types or bowel preparation. True comparison and advantage of the LIFT procedure may not be clear until larger prospective, randomized studies are performed. However, with the current reported data in consideration, we believe that the LIFT procedure is a safe and more effective technique with minimal tissue injury and low recurrence rates.

## Figures and Tables

**Table 1:** Continence Grading Scale of preoperation.

Type of incontinence	Never (CCF-FI = 0)	Rarely (CCF-FI = 1)	Sometimes (CCF-FI = 2)	Usually (CCF-FI = 3)	Always (CCF-FI = 4)
Gas	48	0	4	2	1
Liquid	50	1	3	1	0
Solid	55	0	0	0	0
Wears pad	53	0	0	2	0
Lifestyle alteration	51	0	2	2	0

0 = perfect. 20 = complete incontinence. Never = 0 (never). Rarely = 1/month. Usually = 1/week. Always = ≥1/day. The continence score is determined by adding points from the above table, which takes into account the type and frequency of incontinence and the extent to which it alters the patient's life.

**Table 2:** Demographic and clinical data.

Total number of patients	55

Sex (male/female)	33/20
Median age, y (range)	46 (17–62)
Comorbidities (*n*)	18
Diabetes mellitus	4
Crohn's disease	6
Tuberculosis	3
Hypertension	4
Surgical procedures per patient before LIFT	2.4
Fistula type	
Transsphincteric fistula	31 (6 horseshoe fistulas)
Intersphincteric fistula	20 (10 horseshoe fistulas)
Rectovaginal fistula	4

**Table 3:** Outcomes of LIFT procedure.

Operation time, median (range)	44 (23–88) minutes

Follow-up period, median	16 months

Median time of healing time, median (range)	4 (2–15) months

Healing rate	60% (33/55)

Recurrence rate	40% (22/55)

Postoperative complications	Anal fissure (1)
	Persistent pain (2)
	Wound infection (1)
	Fecal incontinence (1)
	Gas incontinence (1)

Number of recurrence	Horseshoe fistula (10)
	Transsphincteric fistula (8)
	Intersphincteric fistula (3)
	Rectovaginal fistula (1)

Length of stay	2 (1–4) day

**Table 4:** Relationship of previous operations with LIFT success.

Number of operations before LIFT	Success	Recurrence	*p* value
1-2	25	7	
>2	8	15	

Total	33	22	0.002

**Table 5:** Worldwide experience with LIFT.

First author	*N*	Healing	Recurrence	Follow-up
Rojanasakul	18	94.40%	—	—
Shanwani	45	82.20%	17.70%	9 (2–16) months
Bleier	29	57%	10.30%	20 (0–58) weeks
Ellis	31	94%	—	15 (12–30) months
Ooi	25	68%	28%	22 (3–43) weeks
Aboulian	25	68%	12%	27 (8–158) weeks
Sileri	18	83%	NA	6 (4–10) months
Tan	93	86%	6.50%	6 (1–85) weeks
Mushaya	25	68%	8%	16.4 (8.4–31.3) months
Han	21	95%	—	14 (12–15) months
Lo	25	89%	0	9.8 (1–21.5) months
Wallin	93	40%	26%	19 (44–55) months
van Onkelen	41	51%	—	15 (7–21) months
Abcarian	40	74%	8%	18 (2–64) weeks
van Onkelen	22	82%	—	19.5 (3–35) months
Lehmann	17	65%	—	13.5 (8–26) months
Sirikurnpiboon	41	83%	19%	18 weeks
Tan	16	68.80%	—	26 (12–51) months
Liu	38	61%	—	26 (3–44) months
Gingold	15	67%	58%	11.2 (3–15) months
Romaniszyn	14	57%	7.10%	8 (7–17) months
Dalbem	22	77.00%	23%	14 (4–24) months
Tomiyoshi	8	88%	—	2–6 weeks
Baharudin	56	71%	5.35%	20.98 (2.9–151.74) weeks
Ye	43	87%	12.80%	15 (12–24) months
Schulze	75	88%	12%	14.6 ± 1.7 months
Parthasarathi	167	94.10%	5.90%	12 (4–22) months
